# Response to Electronic Health Record Patient Portal–Based Clinical Study Invitations

**DOI:** 10.1001/jamanetworkopen.2025.31624

**Published:** 2025-09-15

**Authors:** Ann Marie Navar, Kathleen Esselink, Ildiko Lingvay, Mereeja Varghese, Hong Li, F. Aaron Brunson, Robert W. Turer, Eric D. Peterson

**Affiliations:** 1 Department of Internal Medicine, University of Texas Southwestern Medical Center, Dallas; 2 Office of Clinical Research, University of Texas Southwestern Medical Center, Dallas; 3 Department of Emergency Medicine, University of Texas Southwestern Medical Center, Dallas

## Abstract

This quality improvement study evaluates demographic factors associated with responses to electronic health record (EHR) recruitment messages for clinical trials.

## Introduction

Clinical trials often struggle to efficiently recruit representative populations.^1,2^ The electronic health record (EHR) and patient portals can facilitate identification and recruitment of potentially eligible study participants via secure portal messages. Demographic factors associated with uptake of this form of digital recruitment remain uncertain.

## Methods

University of Texas Southwestern (UTSW), a quarternary academic medical center, has employed its EHR, MyChart (Epic Systems Co), to identify and invite potentially eligible study participants since 2022. Adult patients with a portal account (614 110 adults [65% of active patients]) can be contacted for research unless they have explicitly opted out. Messages are sent, in bulk, by the centralized recruitment office to potentially eligible patients in their portal that describes the study, indicates their potential eligibility, and asks them to click a button if they are interested in being contacted by the study team. An email or phone notification informs participants of a new research study opportunity in their portal.

In this quality improvement study, we evaluated characteristics of individuals who viewed recruitment messages, who indicated interest in the study, and who were enrolled for all clinical studies using portal messaging from January 2022 to December 2024. Enrollment data were available from our institutional clinical trial management system for all but 3 trials. This analysis used anonymous data tracked as part of ongoing recruitment quality assessment and was determined not human participants research by the UTSW institutional review board.

Participant-level multivariable, mixed-effects logistic regression models were used to evaluate the association between age, sex, race, and ethnicity (as documented in the EHR) and the odds of viewing the message, indicating interest (if viewed), and enrolling in the study, with a random effect to account for study-level variation. A 2-sided *P* value less than 0.05 was used to assess for stastical significance.

## Results

Across 23 studies, recruitment messages were sent to 84 062 individuals (36 109 [43.0%] female; 3068 [3.7%] Asian, 15 947 [19.0%] Black, 7990 [9.5%] Hispanic, and 52 640 [62.6%] White; median [IQR] age 62.5 [55.5-70.6] years). Overall, 29 231 (34.8%) viewed the message, of whom 6237 (21.3%) indicated interest (x of those sent a message [7.4%]). For trials with enrollment data, 1213 were enrolled, corresponding to 1213 of 6168 (19.7%) of those interested and 1213 of 82 066 (1.2%) of all those originally sent a message. Differences in view rates, interest, and enrollment were seen by sex, age, race, and ethnicity (Figure). In multivariable modeling, male sex, younger age, Hispanic ethnicity, Black race, and Asian race were associated with lower likelihood of viewing the message. Asian race and older age, but not Black race or Hispanic ethnicity, were associated with decreased odds of interest in participation among those viewed. Among those interested in participating, both male sex and Black race were associated with decreased odds of enrollment. Overall, among those sent messages, male sex, Hispanic ethnicity, and Black race were associated with decreased odds of overall enrollment.

**Figure.**
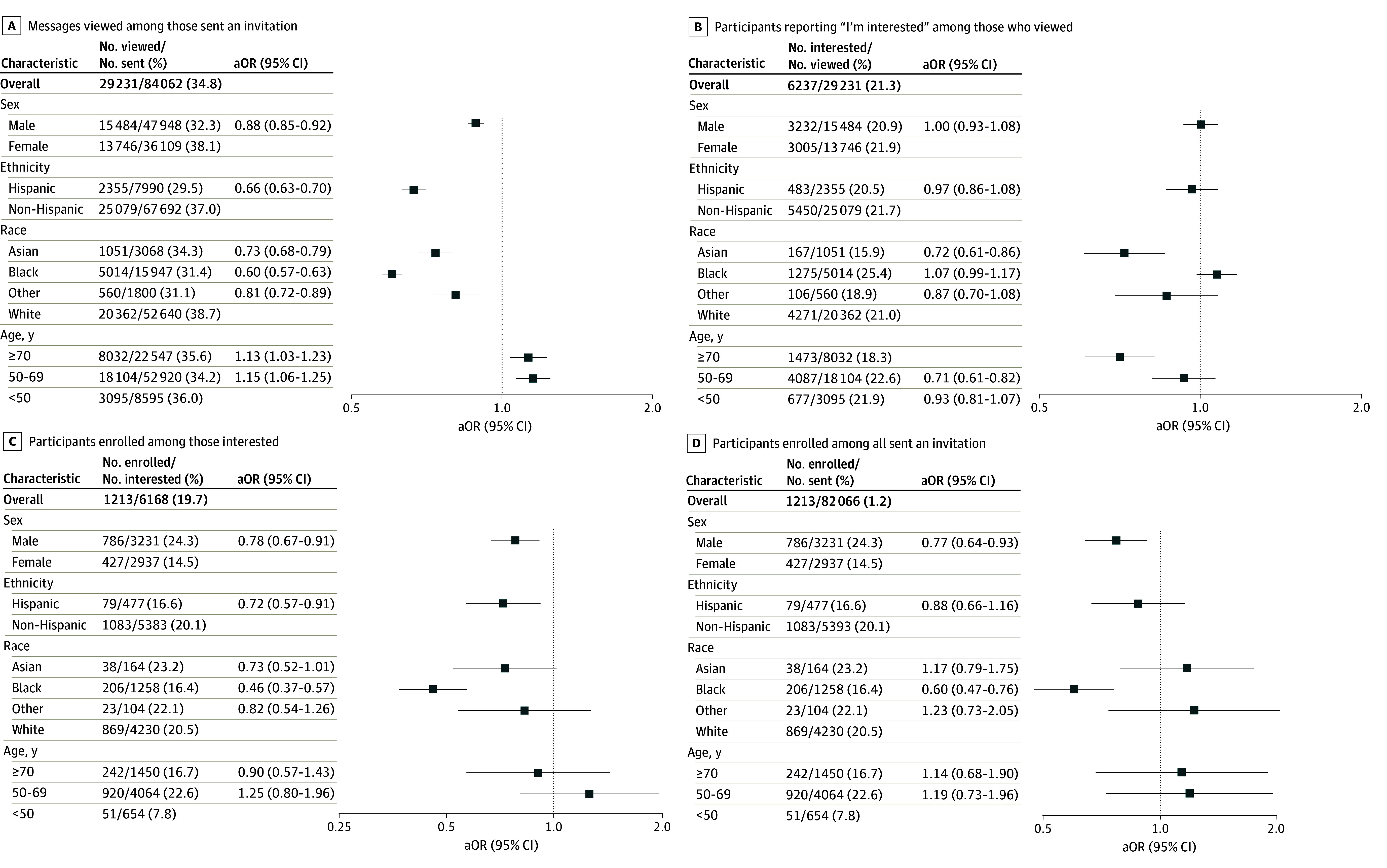
Sex, Race, Ethnicity, and Age and Engagement With Electronic Health Record–Based Clinical Study Recruitment Figure shows multivariable aORs. Panels C and D reflect enrollment for studies where enrollment data were available. aOR indicates adjusted odds ratio.

## Discussion

EHR-based participant identification and recruitment through portal messaging provides an efficient but low-yield method for recruiting potential trial participants. Of those sent a message, only 7% responded with interest, similar to or higher than what was previously reported.^3,4^ EHR-portal based recruitment potentially contributed to disparities due to demographic differences in both who opened their portal message and who was enrolled. Previously reported differences in patient portal utilization by age, race, and ethnicity likely compound this effect.^5^

This study was conducted at a single center with high overall portal utilization, and may not reflect the experience at other centers with lower use. Limitations include lack of socioeconomic or health literacy data.

Importantly, among those who opened their message, Black and Hispanic individuals were similarly interested in participating as other White and non-Hispanic individuals. To help mitigate disparities due to differences in message open rates, individuals from underrepresented demographic groups may be oversampled among those sent recruitment messages. Additional efforts should focus on improving overall portal utilization and the efficiency and equity of enrollment of those who respond as interested.

## Notes

Supplement.
Data Sharing Statement
