# Supplementary material for: Response to Electronic Health Record Patient Portal–Based Clinical Study Invitations
Source: JAMA Netw Open. 2025 Sep 15;8(9):e2531624. doi: 10.1001/jamanetworkopen.2025.31624 (PMC12439056; doi:10.1001/jamanetworkopen.2025.31624)
Supplement: Supplement. — Data Sharing Statement [file jamanetwopen-e2531624-s001.pdf]

Data Sharing Statement

Navar. Response to Electronic Health Record Patient Portal–Based Clinical Study Invitations. *JAMA Netw Open*. Published September 15, 2025. doi:10.1001/jamanetworkopen.2025.31624

Data
Data available: No

Additional Information
Explanation for why data not available: Data for this study include patient information that we do not have permission to disclose externally even if anonymous. Summary data can be shared upon reasonable request to the corresponding author.
